# Giant exchange coupling and field-induced slow relaxation of magnetization in Gd_2_@C_79_N with a single-electron Gd–Gd bond†
†Electronic supplementary information (ESI) available: Additional experimental details, HPLC separation, simulation. See DOI: 10.1039/c8cc00112j


**DOI:** 10.1039/c8cc00112j

**Published:** 2018-03-02

**Authors:** G. Velkos, D. S. Krylov, K. Kirkpatrick, X. Liu, L. Spree, A. U. B. Wolter, B. Büchner, H. C. Dorn, A. A. Popov

**Affiliations:** a Leibniz Institute for Solid State and Materials Research , Dresden 01069 , Germany . Email: a.popov@ifw-dresden.de; b Department of Chemistry , Virginia Polytechnic Institute and State University , Blacksburg , Virginia 24061 , USA; c Virginia Tech Carilion Research Institute , Roanoke , Virginia 24016 , USA . Email: hdorn@vt.edu

## Abstract

Magnetic properties of the azafullerene Gd_2_@C_79_N are studied by SQUID magnetometry. The effective exchange coupling constant *j*_Gd,e_ between the Gd spins and the spin of unpaired electron residing on the single-electron Gd–Gd bond is determined to be 170 ± 10 cm^–1^. Low temperature AC measurements revealed field-induced millisecond-long relaxation of magnetization.

## Abstract 2

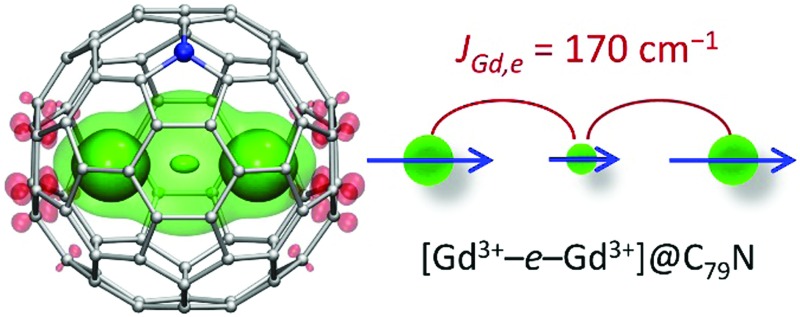
Single-electron Gd–Gd bond in Gd_2_@C_79_N results in giant ferromagnetic coupling between local 4f magnetic moments and unpaired electron spin.

## 

Coupling magnetic atoms into larger clusters is a viable strategy towards molecular magnets. This approach is very successful for transition metals, which can exhibit exchange interactions reaching tens or even hundreds of cm^–1^. In lanthanides, the 4f shells are rather compact, and the overlap of 4f-orbitals with other valence orbitals is very weak. As a result, exchange coupling constants in polynuclear lanthanide compounds rarely exceed 1 cm^–1^. Although even weak interactions strongly affect magnetic properties of polynuclear lanthanide molecular magnets at low temperatures, their vast majority still can be described as a combination of loosely bound spins, rather than a single giant-spin (the concept usually employed for transition metal clusters such as single molecule magnets {Mn_12_} or {Fe_8_}).^1^

The coupling can be enhanced by radical bridges. An exchange coupling of lanthanides with organic radicals (*J*_Ln,R_) can reach several cm^–1^.^2^ The largest *J*_Gd,R_ values‡
‡We use –2*J*_12_*S*_1_*S*_2_ formalism to describe exchange interactions; in the –*J*_12_*S*_1_*S*_2_ formalism, which is also often used, the *J*_12_ values are twice larger. were reported in Gd-nitroxide compounds (6.2 cm^–1^ in ref. 3*a* and –6.0 cm^–1^ in ref. 3*b*), and dinuclear Gd complexes with bridging radicals (–10 cm^–1^ for bipyrimidyl and –27 cm^–1^ for N_2_^3–^).^4^ Yet, the lanthanide-radical couplings exceeding 5 cm^–1^ are rare and are usually considered as very strong.

Direct bonding between lanthanide atoms can potentially lead to much stronger coupling of their magnetic moments. Lanthanides are not known to form Ln–Ln bonds in molecular compounds, but carbon cages can stabilize otherwise non-existent species^5^ and enable formation of dimetallofullerenes (di-EMFs) with covalent Ln–Ln bonds in encapsulated metal dimers.^6^ A stabilization of single-electron metal–metal bonds in di-EMFs is well described for the fullerene C_80_-*I*_h_. This cage usually acts as an acceptor of six electrons, which are transferred to the fullerene orbitals from metals, and forms closed-shell di-EMFs with early lanthanides. However, starting from the middle of the lanthanide row, the Ln_2_ dimers give only 5 electrons to C_80_-*I*_h_, leaving one electron on the Ln–Ln bonding orbital.^7^ Such Ln_2_@C_80_ molecules are not stable because of the open-shell electronic structure of the fullerene. Their stable forms can be obtained by addition of a surplus electron,7*a* quenching the unpaired spin on the cage by an organic radical,6f,^8^ or by substitution of one carbon atom with nitrogen giving azafullerenes Ln_2_@C_79_N (Fig. 1).6a,b Once the fullerene cage is stabilized, these di-EMFs can be very stable molecules despite the presence of the single-electron metal–metal bond.

**Fig. 1:**
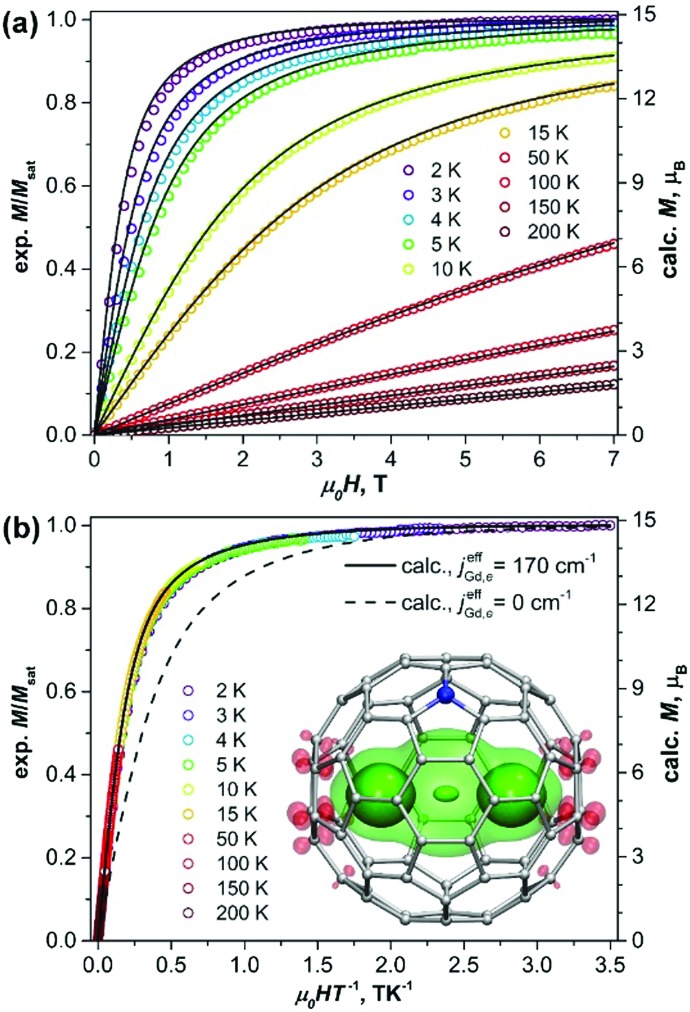
(a) Magnetization curves of Gd_2_@C_79_N measured at different temperatures. (b) Magnetization *versus* the quotient *μ*_0_*HT*^–1^. Dots are experimental points, solid lines are simulations using eqn (1) and the *j*effGd,e value of 170 cm^–1^, the dash line in (b) is a simulation for the [Gd^3+^–e–Gd^3+^] system with non-interacting spins. The inset in (b) shows molecular structure and DFT-computed spin density distribution in Gd_2_@C_79_N (see footnote §) visualized with isovalues ±0.015 a.u. (solid) and ±0.0012 a.u. (semi-transparent). Three well-seen maxima of the spin density correspond to Gd atoms and unpaired electron spin (see also Fig. S3, ESI†).

Magnetic interactions in di-EMFs featuring single-electron Gd–Gd bonds can be formally described by a three-center system [Gd^3+^–e–Gd^3+^] (see Fig. 1 for the spin-density distribution in Gd_2_@C_79_N [see footnote §
§PBE0/TZ2P-DKH level, Orca code.^18^]) with the spin Hamiltonian:1
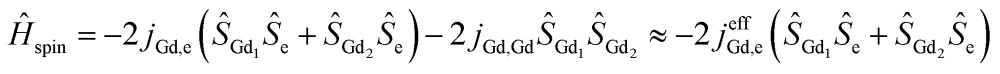



DFT computational studies showed giant ferromagnetic (FM) coupling between localized Gd spins and the unpaired electron spin with *j*_Gd,e_ values of 177 cm^–1^ in Gd_2_@C_80_^–^,^9^ 181–184 cm^–1^ in Gd_2_@C_80_(CH_2_Ph),6*f* and 200 cm^–1^ in Gd_2_@C_79_N.^10^ The Gd–Gd coupling is antiferromagnetic and weak, on the order of –1 cm^–1^ or less, and its neglect gives the approximate form of eqn (1) with the effective coupling constant *j*effGd,e. Thus, theory predicts that the lanthanide-radical coupling in di-EMFs is huge and is much larger than in any other lanthanide-radical compound studied so far. An EPR spectroscopic study of Gd_2_@C_79_N revealed the ground state with the spin *S* = 15/2,6*a* proving the FM coupling of all individual spins. The Dy-electron exchange coupling constant of *j*effDy,e = 32 cm^–1^ was determined experimentally in Dy_2_@C_80_(CH_2_Ph), a single molecule magnet with a high blocking temperature of magnetization.6*f* Here, we report on the static and dynamic magnetic properties of Gd_2_@C_79_N (see footnote ¶
¶Gd_2_@C_79_N was isolated from commercial Gd_3_N@C_80_ (95% purity, Luna Innovations). The major by-product of this commercial sample is Gd_2_@C_79_N (∼3–5%). Gd_2_@C_79_N transfers only 5 electrons to the fullerene cage; whereas, Gd_3_N@C_80_ transfers 6 electrons. This provides a significant chromatographic retention difference between these two EMFs since the pentabromobenzyl (PBB) chromatographic stationary phase is sensitive to the fullerene carbon cage number and the number of electrons transferred from the internal cluster.^17^ With a 1 : 1 mixture of toluene/ortho-dichlorobenzene as the chromatographic solvent system for the PBB chromatographic phase ∼1 mg of Gd_2_@C_79_N was purified from 100 mg of Gd_3_N@C_80_. See ESI† for further details.) and analysis of the exchange coupling. When this manuscript was completed, Gao *et al.* reported the study of the quantum coherence in Gd_2_@C_79_N and determined the *j*_Gd,e_ value of 175 ± 10 cm^–1^, which is very close to the results of this work discussed below.^11^

Fig. 1 shows magnetization curves of Gd_2_@C_79_N measured at different temperatures. The compound exhibits typical paramagnetic behavior. The plot of the magnetization *versus* the quotient *H*/*T* (Fig. 1b) shows that the data measured at different temperatures overlays on a single curve, and only the lowest-temperature points deviate slightly. This proves that Gd_2_@C_79_N has very low magnetic anisotropy.

Fig. 2 plots the product *χ*·*T* measured at different temperatures in different constant fields. At low temperatures the *χ*·*T* values quickly reach the maximum (the temperature of the maximum depends on the magnetic field), then remain constant up to 50–100 K, followed by a slow almost linear decrease at higher temperatures. At 300 K, *χ*·*T* values drop to ca 90% of their 100 K counterparts (absolute values of *χ*·*T* cannot be determined precisely with the low mass of the fullerene available for the measurements). Such a temperature dependence of *χ*·*T* corresponds to the slowly decreasing magnetic moment, which is consistent with the large coupling predicted for Gd_2_@C_79_N.

**Fig. 2:**
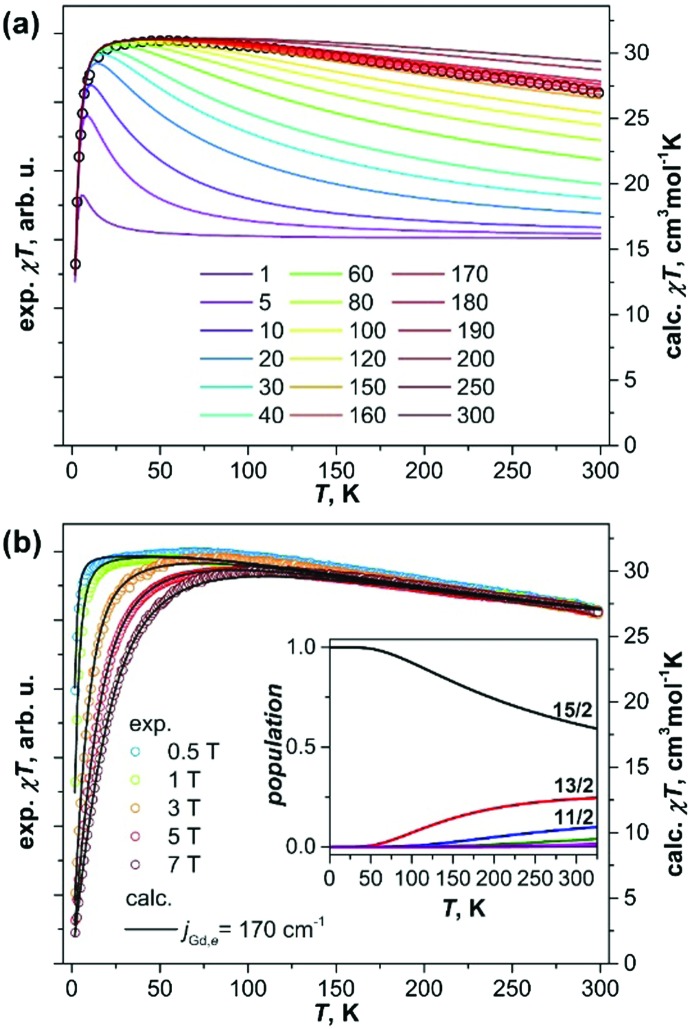
(a) The product *χ*·*T* (*χ* is magnetic susceptibility) measured for Gd_2_@C_79_N in the field of 1 T (dots) and compared to the simulations with different values of the exchange coupling constant *j*effGd,e (coloured lines; the values of *j*effGd,e are given in cm^–1^). (b) Comparison of experimental *χ*·*T* curves measured in different magnetic fields from 0.5 to 7 T (dots) to the results of simulations with the *j*effGd,e constant of 170 cm^–1^. The inset in (b) shows thermal populations of the giant-spin states, in particular *S* = 15/2 (black), 13/2 (red), and 11/2 (blue).

To estimate the constant *j*effGd,e, we simulated *χ*·*T* curves for the [Gd^3+^–e–Gd^3+^] system^12^ using the approximate Hamiltonian in eqn (1) with addition of Zeeman term, magnetic susceptibility was computed using exact fundamental equation for molecular magnetism. The *g*-factor of 1.978 and the positive sign of *j*effGd,e are adopted from the EPR measurements,6*a* and the *j*effGd,e values are varied from 1 to 300 cm^–1^. When *j*effGd,e is small, the *χ*·*T* curves have a sharp peak at low temperature with a fast decay at higher temperature to the *χ*·*T* limit of the non-coupled system (16 cm^3^ mol^–1^ K). With the increase of *j*effGd,e, the peak is growing and becomes less sharp, whereas the higher-temperature decay becomes less steep. It means, the temperature range in which the fully coupled spin system (*χ*·*T* = 31 cm^3^ mol^–1^ K) has the dominant contribution is increasing with *j*effGd,e. Likewise, the decay of *χ*·*T*, caused by a thermal population of the lower-spin states, becomes more gradual because the gap between the high-spin ground state and lower-spin excited states is also increasing. The experimental *χ*·*T* curve in the field of 1 T agrees well with the curves simulated for the *j*effGd,e values in the range of 160–180 cm^–1^ (Fig. 2a), in good agreement with DFT-predictions and recent report by Gao *et al.*^11^ More precise determination of the *j*effGd,e constant is hardly possible because the variation of the computed curves within the interval is comparable to the experimental uncertainties. The constant of *j*effGd,e = 170 cm^–1^ was then used to simulate *χ*·*T* curves measured in different fields (Fig. 2b) as well as to calculate magnetization curves. For both sets of data, very good agreement between experiment and theory is obtained (Fig. 1 and 2).

The spectrum of the approximate Hamiltonian in eqn (1) produced with the *j*effGd,e constant of 170 cm^–1^ spans the energy range of 15*j*effGd,e = 2550 cm^–1^. The eigenstates are grouped into 15 “giant-spin” states with *S* = 15/2, 13/2,…,1/2, 1/2, 3/2,…,13/2. The gaps between the states are all equal *j*effGd,e except for the two *S* = 1/2 states with the energy gap of 2*j*effGd,e. The states within each manifold are (2*S* + 1)-degenerate, but magnetic field lifts the degeneracy, and the 15-line EPR spectrum observed at low temperature6*a* corresponds to transitions within the *S* = 15/2 manifold with weak zero-field splitting. The inset in Fig. 2b shows temperature dependence of the spin populations. The only state to be considered below 50 K is the *S* = 15/2, hence the giant-spin approximation is valid at these temperatures. Magnetization curves computed using eqn (1) and for a single spin *S* = 15/2 show small deviations only above 100 K (Fig. S4, ESI†). The *S* = 13/2 manifold gains significant population above 50 K, and the *S* = 11/2 state should be also considered above 150 K, although the ground state is still the dominant one (>60%) up to room temperature. Thus, in the experimentally relevant temperature range, magnetic properties of Gd_2_@C_79_N are essentially determined by the *S* = 15/2, *S* = 13/2, and to a lesser extent *S* = 11/2 manifolds and their relative populations.

 Eqn (1) does not take into account magnetic anisotropy, but the EPR measurements revealed very small ZFS parameter *D* of 70 mT.6a,^11^ Such a small value cannot be resolved in magnetization data (Fig. S5, ESI†), and thus cannot influence the *j*effGd,e value. More important is the effect of the Gd–Gd coupling. The spectrum of the exact Hamiltonian in the eqn (1) computed with the constants *j*_Gd,Gd_ = –1 cm^–1^ and *j*_Gd,e_ = 170 cm^–1^ is similar to that of the approximate version with *j*effGd,e = 170 cm^–1^, but the energy gaps between the spin states are reduced (Fig. S6, ESI†). The energies of the *S* = 13/2 and *S* = 11/2 states have become *j*_Gd,e_ + 14*j*_Gd,Gd_ and 2*j*_Gd,e_ + 26*j*_Gd,Gd_, respectively, instead of *j*effGd,e and 2*j*effGd,e. Since the *χ*·*T* values are affected by the populations of only two-three lowest energy states, we conclude that the effective coupling constant estimated from the *χ*·*T* curves is related to the *j*_Gd,e_ value as *j*effGd,e ≈ *j*_Gd,e_ + 14*j*_Gd,Gd_. If the DFT-predicted negative sign of the *j*_Gd,Gd_ constant is correct, the real exchange coupling between Gd spins and the unpaired spin residing on the Gd–Gd bonding orbital is even larger than 170 cm^–1^. The structure of the Hamiltonian spectrum implies that when *j*_Gd,e_ is large, and *j*_Gd,Gd_ is too small to induce strong changes in the order of the energy levels as it is the case for Gd_2_@C_79_N, the fitting of *j*_Gd,e_ and *j*_Gd,Gd_ independently would be an ill-defined problem. The [Gd^3+^–N_2_^3–^–Gd^3+^] complex with inner-sphere K^+^ ion is an example of the situation when two parameters can be determined independently.4*c* With the *j*_Gd,R_ and *j*_Gd,Gd_ constants of –27 and –2 cm^–1^, respectively, the effect of the antiferromagnetic Gd–Gd superexchange is sufficient to strongly alter the order of the spin states.

Although Gd is isotropic, millisecond-long field-induced relaxation of magnetization has been observed in some of its salt, single-chain magnets, and molecular magnets.^13^ Our AC susceptibility measurements showed that near 2 K and in the presence of the magnetic field, Gd_2_@C_79_N gives a signal in the out-of-phase susceptibility *χ*′′ (Fig. 3). Fig. 3a shows *χ*′′ curves obtained at 1.8 K with various DC magnetic fields between 0 and 0.8 T. Zero-field measurements did not give detectable *χ*′′ responses, but the peak emerged when the field of 0.1 T was applied. Its amplitude grows with the field till the maximum at 0.3 T, and then decreases again at higher fields. Fitting of the data with the generalized Debye model (Fig. 3b) gave relaxation times (*τ*_m_), varying from 8 ms at 0.1 T to 18–22 ms in the field of 0.4–0.8 T (Fig. 3c).

**Fig. 3:**
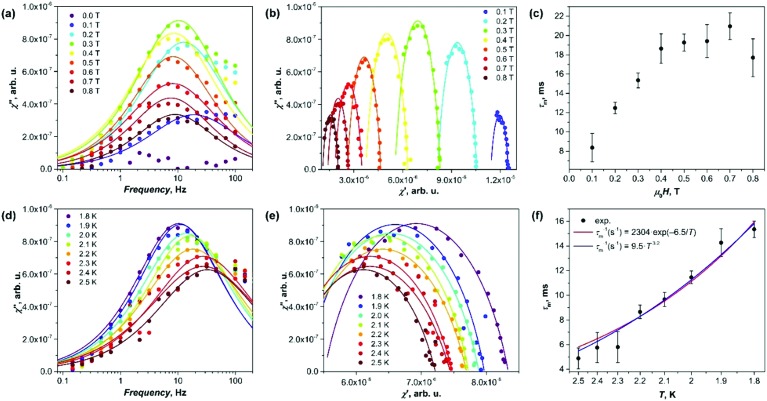
AC magnetometry studies of Gd_2_@C_79_N. (a) Out-of-phase susceptibility *χ*′′ measured at 1.8 K in different constant DC fields. (b) Same as (a), but showing the out-of-phase signal susceptibility *χ*′′ *versus* in-phase susceptibility *χ*′ (Cole–Cole plots). (c) Relaxation times of magnetization as a function of the magnetic field. (d) Out-of-phase susceptibility *χ*′′ measured at different temperatures in the constant field of 0.3 T. (e) Same as (d), but showing the Cole–Cole plots. (f) Relaxation times of magnetization in the field of 0.3 T as a function of temperature; the lines are fits to the Orbach relaxation mechanism (red, *U*^eff^ = 6.5 K) or to the power law (blue, *n* = 3.2). The dots in (a,b,d and e) are experimental values, lines are the fits with generalized Debye model, the latter is also used to determine relaxation time from AC data.

The temperature dependence was measured in the field of 0.3 T between 1.8 and 2.5 K (Fig. 3d–f; the signal could not be measured reliably at higher temperature due to the amplifier disturbing the low-intensity signals at frequencies above 100 Hz). The relaxation times dropped from 16 ms at 1.8 K to 5 ms at 2.5 K. The temperature dependence of *τ*_m_ is shown in Fig. 3f with both exponential and power law fits. Both functions give a comparable agreement. The exponential dependence *τ*_m_^–1^ = *τ*_0_^–1^ exp(–*U*^eff^/*T*) corresponds to the Orbach relaxation mechanism with the barrier *U*^eff^ = 6.5 ± 0.5 K and *τ*_0_ = 4 ± 1 × 10^–4^ s. This *U*^eff^ value is larger than zero-field splitting of the *S* = 15/2 manifold estimated from EPR data (∼3 K), and Zeeman splitting in the field of 0.3 T (∼2.3 K).

The fit of experimental relaxation rates fitted with the power function *τ*_m_^–1^ ∼ *AT*^*n*^ gives the *n* value of 3.2 ± 0.2 (Fig. 3f). At low *T*, relaxation often follows the direct mechanism (*τ*_m_^–1^ ∼ *T*), in which the spin flip is accompanied by the emission/absorption of the phonon with the frequency, matching the splitting of the spin levels.^14^ However, if the number of spins is much larger than the number of resonant phonons, the energy dissipation is hampered resulting in a phonon bottleneck,^15^ which elongates the relaxation with complex temperature dependence (*τ*_m_^–1^ ∼ *T*^*b*^, 1 ≤ *b* ≤ 4).^16^ Finally, the Raman mechanism with *τ*_m_^–1^ ∼ *T*^9^ dependence for Kramers systems is plausible at higher temperatures.^14^ Thus, the value of *n* = 3.2 determined for Gd_2_@C_79_N may be an indication of the bottlenecked direct relaxation mechanism near 2 K, but in the view of significant uncertainties of the values this conclusion remains tentative. Earlier, the phonon bottleneck was often recognized as the most plausible reason for a long relaxation in several other Gd compounds.13b–f

To conclude, the giant exchange coupling between the localized 4f-spins of Gd and the spin of the unpaired electron residing on the Gd–Gd bond is found in Gd_2_@C_79_N. The exchange coupling constant *j*effGd,e of 170 cm^–1^ is the largest constant ever observed for molecular lanthanide compounds. The inner space of the fullerene provides the perfect environment for unprecedented spin states of lanthanides.

The authors acknowledge the European Research Council under the European Union's Horizon 2020 programme (Grant No. 648295 “GraM3”) and Deutsche Forschungsgemeinschaft (grant PO 1602/4-1). K. Kirkpatrick acknowledges financial support from a Luther & Alice Hamlett Scholarship.

## Conflicts of interest

There are no conflicts to declare.

## Supplementary Material

Supplementary information

## References

[cit1] Sessoli R., Gatteschi D., Caneschi A., Novak M. A. (1993). Nature.

[cit2] Demir S., Jeon I.-R., Long J. R., Harris T. D. (2015). Coord. Chem. Rev..

[cit3] Kanetomo T., Yoshitake T., Ishida T. (2016). Inorg. Chem..

[cit4] Demir S., Zadrozny J. M., Nippe M., Long J. R. (2012). J. Am. Chem. Soc..

[cit5] Popov A. A., Yang S., Dunsch L. (2013). Chem. Rev..

[cit6] Fu W., Zhang J., Fuhrer T., Champion H., Furukawa K., Kato T., Mahaney J. E., Burke B. G., Williams K. A., Walker K., Dixon C., Ge J., Shu C., Harich K., Dorn H. C. (2011). J. Am. Chem. Soc..

[cit7] Velloth A., Imamura Y., Kodama T., Hada M. (2017). J. Phys. Chem. C.

[cit8] Kareev I. E., Bubnov V. P., Yagubskii E. B. (2008). Russ. Chem. Bull..

[cit9] Mansikkamäki A., Popov A. A., Deng Q., Iwahara N., Chibotaru L. F. (2017). J. Chem. Phys..

[cit10] Cimpoesu F., Frecus B., Oprea C. I., Ramanantoanina H., Urland W., Daul C. (2015). Mol. Phys..

[cit11] Hu Z., Dong B.-W., Liu Z., Liu J.-J., Su J., Yu C., Xiong J., Shi D.-E., Wang Y., Wang B.-W., Ardavan A., Shi Z., Jiang S.-D., Gao S. (2018). J. Am. Chem. Soc..

[cit12] PHI code: Chilton N. F. Anderson R. P. Turner L. D. Soncini A. Murray K. S., J. Comput. Chem., 2013, 34 , 1164 . 23386394 10.1002/jcc.23234

[cit13] Davis C. F., Strandberg M. W. P., Kyhl R. L. (1958). Phys. Rev..

[cit14] Orbach R. (1961). Proc. R. Soc. A.

[cit15] Van Vleck J. H. (1941). Phys. Rev..

[cit16] Bartolomé E., Arauzo A., Luzón J., Bartolomé J. and Bartolomé F., in Handbook of Magnetic Materials, ed. E. Brück, Elsevier, 2017, p. 1.

[cit17] Liu X., Zuo T., Dorn H. C. (2017). J. Phys. Chem. C.

[cit18] Neese F. (2012). Wiley Interdiscip. Rev.: Comput. Mol. Sci..

