# Supplementary material for: Giant exchange coupling and field-induced slow relaxation of magnetization in Gd2@C79N with a single-electron Gd–Gd bond
Source: Chem Commun (Camb). 2018 Mar 2;54(23):2902–5. doi: 10.1039/c8cc00112j (PMC5885278; doi:10.1039/c8cc00112j)
Supplement: Supplementary file 1 [file CC-054-C8CC00112J-s001.pdf]

Electronic Supplementary Material (ESI) for ChemComm.
This journal is © The Royal Society of Chemistry 2018

Giant exchange coupling and slow relaxation of magnetization in $Gd_2@C_{79}N$ with a single-electron Gd–Gd bond

G. Velkos,[a] D. S. Krylov,[a] K. Kirkpatrick,[b] X. Liu,[b] L. Spree,[a] A. U. B. Wolter,[a] B. Büchner,[a] H. C. Dorn*[bc] and A. A. Popov*[a]

Supporting Information

Separation and characterization of $Gd_2@C_{79}N$ **S2**

Additional information on the spin density distribution **S3**

Simulations of magnetization curves **S4**

Spectrum of the spin Hamiltonian **S5**

Magnetization relaxation times determined from AC measurements **S6**

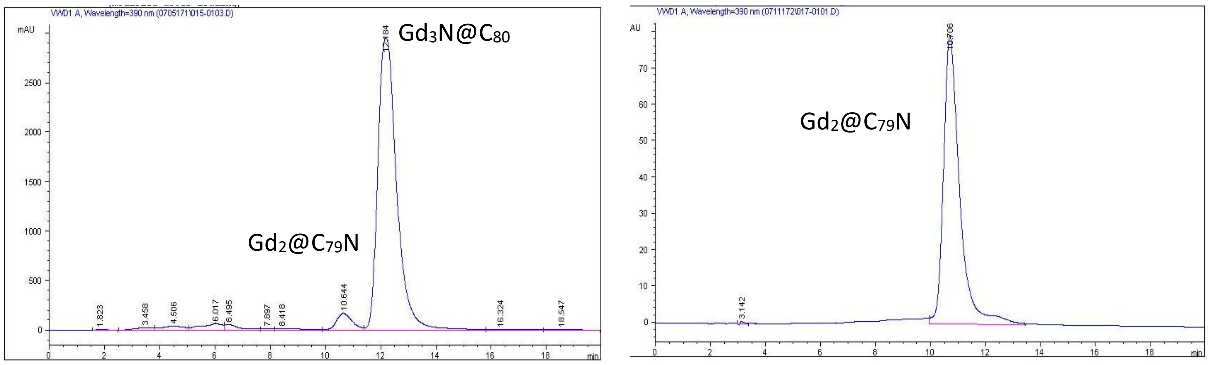

Figure S1 *HPLC traces of commercially available $Gd_3N@C_{80}$ (left) and isolated $Gd_2@C_{79}N$ (right). PBB column (4.5 mm I.D. x 250 mm); λ = 390 nm; flow rate 1.0 mL/min; 1:1 toluene: o-dichlorobenzene as eluent; 25 ℃; 100 uL injection.*

After the first step purification with PBB column, the sample was subjected to recycling HPLC at the Buckyprep column to remove the traces of $Gd_3N@C_{80}$ and other fullerenes potentially present in the sample. The mass spectrum obtained after the second purification step (Figure S2) confirms the high purity of isolated $Gd_2@C_{79}N$.

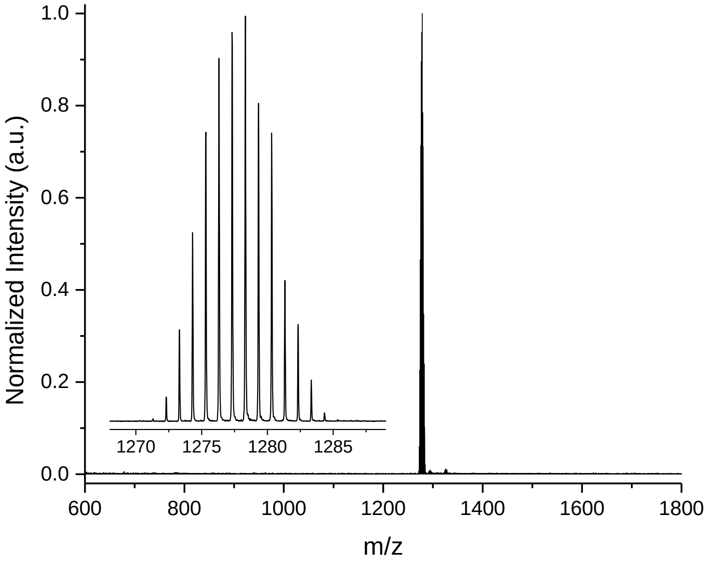

Figure S2. MALDI mass spectrum of purified $Gd_2@C_{79}N$ negative ion mode. The inset show isotopic distribution pattern of the signal.

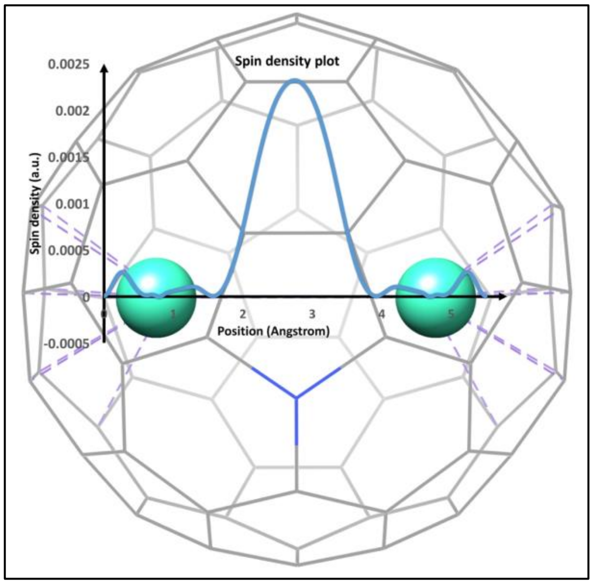

Figure S3. Spin density distribution in the $Gd_2@C_{79}N$ a computed at the B3LYP level with 6-31G(d) basis set for C and N and ECP-121G basis for Gd. The X axis indicates the position between two Gd atoms and Y axis shows spin density for each position. Blue shows the N atom on $C_{79}N$ cage. The distance between two Gd atoms is 3.90 angstrom. Effective core potential for Gd used in these calculations includes 4f-electrons, so that the calculation emphasizes only the valence part of the spin density, which is presented by the unpaired electron residing on the Gd-Gd bonding MO. In contrast to this, Figure 1 in the manuscript shows the spin density computed with the full-electron basis set, which therefore has contributions from both 4f electrons as well as from the unpaired spin.

Magnetic properties of $Gd_2@C_{79}N$

DC magnetometry measurements were performed with VSM-SQUID system MPMS 3, field sweep rate 5.6 mT/s, temperature sweep rate 5K/min. AC magnetometry was performed with MPMS XL system, the amplitude of the oscillating field was 5 Oe.

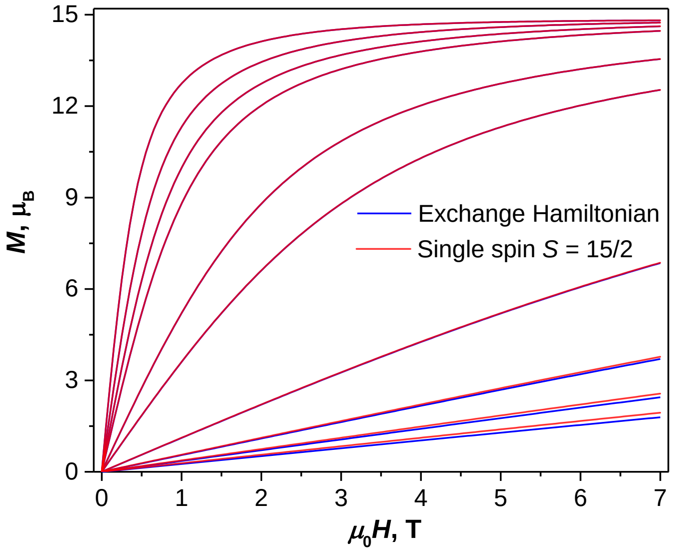

Figure S4. Comparison of the magnetization curves simulated using the spin Hamiltonian: $\hat{H}_{\text{spin}} = -2 j_{\text{Gd},e}^{\text{eff}} (\hat{S}_{\text{Gd}_1} \cdot \hat{S}_e + \hat{S}_{\text{Gd}_2} \cdot \hat{S}_e)$, $j_{\text{Gd},e}^{\text{eff}}$ = 170 cm^{-1}, and for the single giant spin *S* = 15/2. Temperatures are 2, 3, 4, 5, 10, 15, 50, 100, 150, and 200 K. The small difference between the two types of curves is noticeable above 50 K.

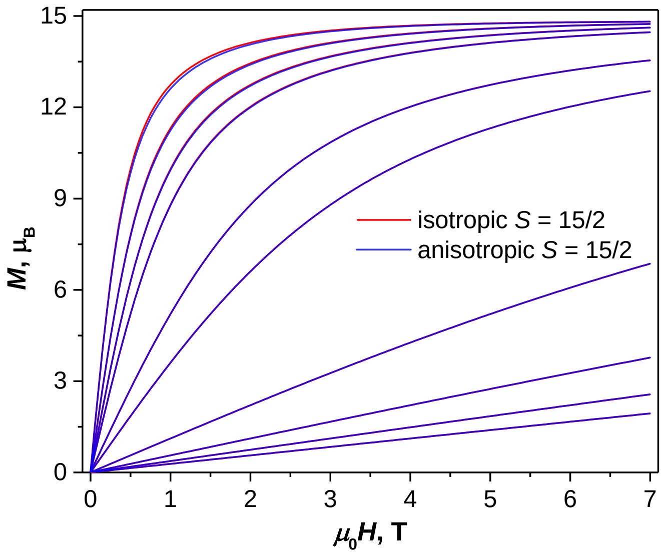

Figure S5. Magnetization curves simulated for the isotropic spin *S* = 15/2, and for the slightly anisotropic spin *S*=15/2 with zero field splitting parameter *D* = 0.01 cm^{-1} inferred from the EPR spectrum of $Gd_2@C_{79}N$. Temperatures are 2, 3, 4, 5, 10, 15, 50, 100, 150, and 200 K. Very small derivation can be seen only at 2 K, at all other temperatures the curves are indistinguishable.

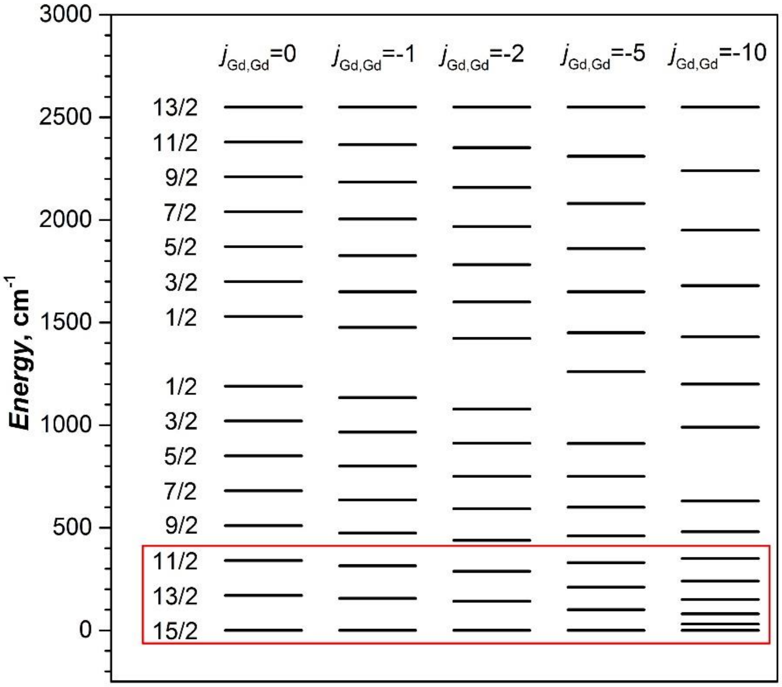

Figure S6. Spectra of the spin Hamiltonian:

$$\hat{H}_{\text{spin}} = -2j_{\text{Gd},e}(\hat{S}_{\text{Gd}_1} \cdot \hat{S}_e + \hat{S}_{\text{Gd}_2} \cdot \hat{S}_e) - 2j_{\text{Gd,Gd}}\hat{S}_{\text{Gd}_1} \cdot \hat{S}_{\text{Gd}_2} \quad \text{(S1)}$$

computed with the $j_{\text{Gd},e}$ = 170 cm^{-1} and different values of $j_{\text{Gd,Gd}}$. The $j_{\text{Gd,Gd}}$ values in the figure are given in cm^{-1}, the left column gives the giant spin value for each manifold. The energies of the two series of the spin states can be computed using the following formulae:

$$\Delta E_i = (15/2 - S_i) \cdot \{j_{\text{Gd},e} + (15/2 + S_i) \cdot j_{\text{Gd,Gd}}\}, \;\; S_i \in [1/2, \ldots, 15/2] \quad \text{(S2)}$$

$$\Delta E_j = (15/2 + 1 + S_j) \cdot \{j_{\text{Gd},e} + (15/2 - 1 - S_j) \cdot j_{\text{Gd,Gd}}\}, \;\; S_j \in [1/2, \ldots, 13/2] \quad \text{(S3)}$$

Red rectangle highlights the states with the reasonable population at room temperature. Thermal population of these states affects the shape of the $\chi \cdot T$ function. As long as $j_{\text{Gd,Gd}}$ remains on the order of −1 cm^{-1}, the decay of the $\chi \cdot T$ function in the 100-300 K range is still mainly determined by the thermal population of the S = 13/2 state (with smaller influence of S = 11/2, see Fig. 2b). Therefore, the effective $j_{\text{Gd},e}^{\text{eff}}$ constant determined from the comparison of the experimental and computed $\chi \cdot T$ values corresponds to the energy difference between these two states (see Eq. S2):

$$j_{\text{Gd},e}^{\text{eff}} \approx E_{13/2} - E_{15/2} = j_{\text{Gd},e} + 14 j_{\text{Gd,Gd}} \quad \text{(S4)}$$

With the increase of the $j_{\text{Gd,Gd}}$ value more and more spin states become accessible in the experimentally relevant temperature range, making the Eq. S4 not valid for large $j_{\text{Gd,Gd}}$ (such as the value of −10 cm^{-1} shown in Fig. S6)

Magnetization relaxation times of $Gd_2@C_{79}N$ determined from AC measurements

Table S1. Relaxation times measured at 1.8 K with different values of the DC field

H, T	τ_m, ms	St. Dev., ms	α
0.1	8.4	1.5	0.29
0.2	12.5	0.6	0.26
0.3	15.4	0.8	0.27
0.4	18.6	1.5	0.27
0.5	19.3	0.9	0.24
0.6	19.4	1.7	0.28
0.7	21.0	1.4	0.23
0.8	17.7	2.0	0.21

Table S2. Relaxation times measured at different temperatures with the DC field of 0.3 T

T, K	τ_m, ms	St. Dev., ms	α
1.8	15.4	0.7	0.27
1.9	14.3	1.1	0.26
2.0	11.5	0.5	0.29
2.1	9.7	0.6	0.28
2.2	8.7	0.6	0.33
2.3	5.8	1.3	0.35
2.4	5.8	1.2	0.39
2.5	4.9	0.9	0.34
